# Synergism and the mechanism of action of the combination of α-mangostin isolated from *Garcinia mangostana* L. and oxacillin against an oxacillin-resistant *Staphylococcus saprophyticus*

**DOI:** 10.1186/s12866-016-0814-4

**Published:** 2016-08-26

**Authors:** Sineewan Phitaktim, Mullika Chomnawang, Kittipot Sirichaiwetchakoon, Benjawan Dunkhunthod, Glyn Hobbs, Griangsak Eumkeb

**Affiliations:** 1 School of Pharmacology, Institute of Science, Suranaree University of Technology, Nakhon Ratchasima, 30000 Thailand; 2 Department of Microbiology, Faculty of Pharmacy, Mahidol University, Rajathevi, Bangkok Thailand; 3 School of Pharmacy and Biomolecular Sciences, Liverpool John Moores University, Byrom Street, Liverpool, L3 3AF UK

**Keywords:** α-mangostin, *Garcinia mangostana*, Oxacillin, Oxacillin-resistant *S. saprophyticus*, Synergistic activity, Mechanism of action

## Abstract

**Background:**

Globally, staphylococci have developed resistance to many antibiotics. New approaches to chemotherapy are needed and one such approach could be to use plant derived actives with conventional antibiotics in a synergestic way. The purpose of this study was to isolate α-mangostin from the mangosteen (*Garcinia mangostana* L.; GML) and investigate antibacterial activity and mechanisms of action when used singly and when combined with oxacillin against oxacillin-resistant *Staphylococcus saprophyticus* (ORSS) strains. The isolated α-mangostin was confirmed by HPLC chromatogram and NMR spectroscopy. The minimum inhibitory concentration (MIC), checkerboard and killing curve were determined. The modes of action of these compounds were also investigated by enzyme assay, transmission electron microscopy (TEM), confocal microscopic images, and cytoplasmic membrane (CM) permeabilization studies.

**Results:**

The MICs of isolated α-mangostin and oxacillin against these strains were 8 and 128 μg/ml, respectively. Checkerboard assays showed the synergistic activity of isolated α-mangostin (2 μg/ml) plus oxacillin (16 μg/ml) at a fractional inhibitory concentration index (FICI) of 0.37. The kill curve assay confirmed that the viability of oxacillin-resistant *Staphylococcus saprophyticus* DMST 27055 (ORSS-27055) was dramatically reduced after exposure to isolated α-mangostin (2 μg/ml) plus oxacillin (16 μg/ml). Enzyme assays demonstrated that isolated α-mangostin had an inhibitory activity against β-lactamase in a dose-dependent manner. TEM results clearly showed that these ORSS-27055 cells treated with this combination caused peptidoglycan and cytoplasmic membrane damage, irregular cell shapes and average cell areas were significantly larger than the control. Clearly, confocal microscopic images confirmed that this combination caused considerable peptidoglycan damage and DNA leakage. In addition, the CM permeability of ORSS-27055 was also increased by this combination of actives.

**Conclusions:**

These findings provide evidence that isolated α-mangostin alone has not only some activity but also shows the synergistic activity with oxacillin against ORSS-27055. The chromone and isoprenyl structures could play a significant role in its action. This synergistic activity may involve three mechanisms of action. Firstly, potential effects of cytoplasmic membrane disruption and increases permeability. Secondly, inhibit β-lactamase activity. Finally, also damage to the peptidoglycan structure. We proposes the potential to develop a novel adjunct phytopharmaceutical to oxacillin for the treatment of ORSS. Future studies require clinical trials to establish if the synergy reported can be translated to animals and humans.

## Background

Antibiotic resistance in staphylococci has been globally documented [1]. The resistance of these strains to board spectraum β-lactam antibiotics, such as methicillin, oxacillin, and flucloxacillin, have emerged rapidly only a few years after the introduction of the first drug in this class and there has been a steady risen in the incidence of methicillin-resistant *S. aureus* (MRSA) in clinical isolates [1, 2]. A previous study of 12 hospitals in Virginia, USA found that overall 53 % of *S. aureus* isolates were resistant to oxacillin [3]. In addition, to pathogenic *S. aureus*, currently *S. saprophyticus,* a coagulase- negative staphylococcus that frequently causes community-associated uncomplicated urinary tract infection (UTI) in young and middle-aged women, has become resistant to β-lactam antibiotics, such as methicillin by acquisition of staphylococcal cassette chromosome *mec* (SCC*mec*) element [4, 5]. These problems present an urgent need to search for new antibiotics and novel approaches to treating these bacterial infections. Plant-derived antimicrobials are a potential source of novel therapeutics because plants are known to produce various antimicrobial molecules to protect themselves from plant or environmental pathogens [6]. Furthermore, drug combination strategies, in particular, phytochemical and antibiotic combination approachs have been recommended in several studies to combat multiple drug-resistant bacteria [7–9]. Mangosteen (the queen of fruit), belonging to the family Guttiferae, is a tropical evergreen tree that is widely cultivated throughout India, Myanmar, Malaysia, Philippines, Sri Lanka, and Thailand [10]. The pericarp (peel, rind, and hull) or the ripe fruit of GML has been traditionally used for the treatment of diarrhea, inflammation, abdominal pain, dysentery, wound infection, suppuration and chronic ulcers [11]. The α-mangostin, a xanthone derivative, has been found to possess several beneficial biological activities, such as a competitive antagonist of the histamine H_1_ receptor and weak antioxidant properties [12], antibacterial activity against *Helicobacter pylori*, anti-inflammatory activities, inhibition of oxidative damage by human low-density lipoproteins (LDL), antimicrobial activity against methicillin-resistant *Staphylococcus aureus* [13]. However, the recent studies have not reported on the antibacterial activity of α-mangostin and synergism with oxacillin against oxacillin-resistant *S. saprophyticus*. To this end, the present study was instigated to elucidate antibacterial and synergistic activity of α-mangostin isolated from the GML pericarp and oxacillin either alone or in combination against this strain. The antibacterial actions and cell line toxicity of these compounds were also investigated.

## Methods

### Plant materials, β-lactam antibiotics, bacterial strains, and cell line

The dried fruit hulls of mangosteen were purchased locally in Nakhon-Ratchasima, Thailand. The samples were identified by Dr. Paul J. Grote, Suraneree University of Technology. The voucher specimens (SGM0804U) were deposited in the School of Pharmacology, Institute of Science, Suranaree University of Technology, Nakhon-Ratchasima, Thailand. The mature fruit was cleaned. The fruit rinds were cut into small pieces, dried in a hot oven at 50 °C for 72 h and ground into powder, passed through a sieve (20 mesh). The powdered sample was kept in an airtight container protected from light until used.

All clinical isolates of *S. saprophyticus* were obtained from the Department of Medical Science, National Institute of Health, Ministry of Public Health, Bangkok, Thailand. The susceptible strain *S. aureus* ATCC 29213, a reference strain, was obtained from the American Type Culture Collection (ATCC). Oxacillin, Nisin, o-nitrophenol-β-D-galactoside (ONPG), α-mangostin standard, and β-lactamase type IV isolated from *E. cloacae* were obtained from Sigma-Aldrich, UK. Meuller-Hinton broth (MHB) and Mueller-Hinton agar (MHA) were purchased from Oxoid (Basingstoke, UK).

The 3T3-L1 mouse embryonic fibroblasts and bovine calf serum (CBS) were purchased from the American Type Culture Collection (ATCC, USA). 3-(4,5-Dimethylthiazol-2-yl)-2,5-diphenyltetrazolium bromide (MTT), Dulbecco’s Modified Eagle’s medium (DMEM), Fetal bovine serum (FBS), Penicillin-Streptomycin, N-2-hydroxyethylpiperazine-N-2-ethane sulfonic acid (HEPES) were obtained from Gibco Invitrogen (Grand Island, NY, USA).

### Isolation and purification of α-mangostin

α-mangostin from the pericarp of GML was isolated and purified according to previous methods with some modifications [12, 14]. Briefly, 1 kg of dried pericarp powder of GML was extracted successively with n-hexane, dichloromethane (CH_2_Cl_2_), ethanol, and acetone using a Soxhlet extractor. The filtered extracts were then concentrated using a rotatory evaporator to provide a yellowish power for n-hexane (84 g) and dichloromethane (106 g), brown sticky semi-solid for ethanol (262 g) and acetone (130 g) respectively.

The CH_2_Cl_2_ crude extract was further separated by silica gel column chromatography to yield 11 fractions. These fractions were subjected to HPLC (C18 column, a mobile phase of methanol-water (85:15) and a flow rate of 1.0 ml/min, a photodiode array detector) and purified using preparative thin layer chromatography to obtain isolated α-mangostin. The α-mangostin was analyzed by HPLC and its chemical structure was elucidated by ^1^H NMR and ^13^C NMR. The spectrum structure data of this compound was compared with those previously reported [15].

### Standardised bacterial suspensions

To select bacterial suspensions with a known viable count, the method of Liu et al. [16] was followed with little modifications. MHA and Cation-adjusted Mueller-Hinton broth (CAMHB) were used as a medium.

### Minimum inhibitory concentration (MIC) and checkerboard determinations

The antibacterial activity and drug interaction of isolated α-mangostin from the pericarp of GML with oxacillin were performed by MIC and checkerboard assays, respectively using broth macrodilution procedure. These assays were conducted following the methods of Clinical and Laboratory Standard Institute guidelines [16, 17]. In summary, 0.25 ml of 5 × 10^6^ cfu/ml bacterial suspensions was added to a series of 2.25 ml CAMHB plus 1 in 10 serial dilutions of the α-mangostin plus oxacillin combinations to give 5x10^5^ cfu/ml. Tubes of the broth without antimicrobialsl were used as the control. The cultures were incubated for 24 h at 37 °C. The tests were carried out in triplicate. MICs were determined for each antibacterial combination and the isobolograms were plotted. The interaction between the two agents was calculated by the fractional inhibitory concentration (FIC) index of the combination. The FIC of each agent was calculated by the complete growth inhibition of microorganism in the combination tube. The following formula was used for FIC index calculation: FIC of α-mangostin = MIC α-mangostin in the combination/MIC of α-mangostin alone; FIC of oxacillin = MIC of oxacillin in the combination/MIC of oxacillin alone; therefore, FIC index = FIC of α-mangostin + FIC of oxacillin. When the FIC index of the combination is equal to or less than 0.5, the combination is defined as synergistic; when the FIC index falls between 0.5 and 4.0, it indicates ‘no interaction’ between the agents and a value above four is considered to show antagonism between the two compounds [18]. *S. aureus* ATCC 29213 was used as positive control. The MICs and FIC index is presented as the median values obtained in duplicates from three independent experiments.

### Kill curve determinations (Viable counts)

The experiment was carried out to confirm antibacterial and synergistic activities of isolated α-mangostin from the pericarp of GML when used singly and in combination with oxacillin as previously described by Mun et al. and Richards et al. [19, 20]. Compounds were used at the half minimal inhibitory concentration (1/2-MICs) when each compound was assessed alone. However, to study the effect of the compounds in combination, each compound was used at the MIC that yielded synergism.

### Enzyme assays

The ability of isolated α-mangostin from the pericarp of GML to inhibit the activity of *β*-lactamase type IV isolated from *E. cloacae* was determined in accordance with the methods of Eumkeb et al. and Richards et al. [21, 22]. Concisely, benzylpenicillin, a substrate for β-lactamase type IV, was adjusted to concentrations sufficient to hydrolyze 50-60 % substrate within 5 min, β-lactamase at 100 μg/ml was used. The α-mangostin at 1, 2, 4 and 8 μg/ml were preincubated with the enzyme in 50 mM sodium phosphate buffer (pH 7.0) at 37 °C for 5 min before adding a substrate. A time - course assay was performed at 0, 5, 10, 15 and 20 min using methanol/acetic acid (100:1) as a stopping agent. Aliquots (10 μl) of each sample were injected onto a reverse-phase HPLC (Ascentis C18 column) to analyse the remaining benzylpenicillin. The mobile phase consisted of 10 mM ammonium acetate (pH 4.5 acetic acid): acetonitrile (75:25) with a flow rate of 1 ml/min, UV detection of peaks was at 200 nm,, and the column maintained at 35 °C. The quantity of remaining benzylpenicillin was calculated by comparing the area under the chromatographic curve.

### Transmission electron microscopy (TEM)

To determine the ultrastructure morphology of bacteria after treatment with isolated α-mangostin from the pericarp of GML either alone or in combination with oxacillin, the method of Richards et al. [22] was followed. To investigate the mechanism of action of these agents, the half-MICs of both compounds used alone and Sub-FICs of the combination, were chosen for examination. To confirm the effects of these agents either used singly and in combination on cell size, the cell area from micrographs were analyzed by measuring cell width multiplied by cell length (nm^2^). The experiment was performed in triplicate, and the cell areas are displayed as mean ± SEM [23].

### Immunofluorescence staining and confocal microscopy

The disruption of peptidoglycan after exposure to α-mangostin either used singly or in conjunction with oxacillin was performed using immunofluorescence and visualized under a confocal laser scanning microscope following the method of Teethaisong et al. [24]. Shortly, after the FIC index was obtained from checkerboard, the half-MICs value of isolated α-mangostin or oxacillin alone and the 3/4 FIC of this combination that showed synergistic FIC index was chosen for examination. The cells grown without any antibacterial agent were employed as control [25].

### Cytoplasmic membrane (CM) permeabilization assays

Two methods were used to assess CM permeabilization. Firstly; the CM permeabilization experiment was performed, with some modifications, to confirm results as previously described by Shen et al. and Zhou et al*.* [26, 27]. Shortly after the FIC index was determined by the checkerboard assay, the half-MIC values for isolated α-mangostin or oxacillin alone, and the 3/4 MIC values for this combination that indicated synergistic FIC index were selected against ORSS to measure CM permeability. This method was performed by measuring the release of UV-absorbing material (Varian’s Cary 100 UV-Vis spectrophotometer, Varian, Inc., California, USA) [21].

Secondly, the α-mangostin-induced permeabilization of the CM of ORSS was determined essentially as recently described [28]. In brief, to assay CM permeabilization, the wells contained 50 μl ONPG plus either half-MIC values for isolated α-mangostin or oxacillin alone and the 3/4 MIC values for this combination that indicated synergistic FIC index were prepared shortly before the experiment. Finally, 50 μl of cell suspension (OD 0.3) was added to the wells to give a final concentration of 100 μg/ml ONPG. After warming to 37 °C the plates were positioned in the plate reader at 37 °C. ONPG uptake and cleavage by β-galactosidase within the cytoplasm was characterized by monitoring absorption over a period of 120 min at 420 nm. Complete permeabilization was induced in the presence of 0.5 μg/ml Nisin as a positive control and wells lacking drugs or isolated α-mangostin test served as a negative control [29, 30].

### In vitro cytotoxicity test (MTT assays)

The 3T3-L1 preadipocytes were cultured in Dulbecco’s modified Eagle’s medium (DMEM) with high glucose, supplemented with 10 % CBS, 1.5 mg/ml sodium bicarbonate, 100 U/ml penicillin and 100 μg/ml streptomycin until confluent. The cells were maintained at 37 °C in 5 % CO_2_ and 95 % humidity. The cytotoxic effect of α-mangostin, oxacillin, either alone or in combination on cell proliferation was determined using a tetrazolium dye (MTT) in a colorimetric assay [31]. Briefly, the cells were seeded in a 96-well plate at a density of 5 × 10^3^ cells/well. The cells were allowed to adhere for 48 h and then were treated with various concentrations of three compounds for 24 h. After incubation, the cultured medium was removed, and 0.5 mg/ml of MTT was added. Then, cells were further incubated for 4 h at 37 °C. Formazan crystals formed by viable cells were dissolved in DMSO and absorbance was measured at 540 nm with a microplate spectrophotometer (Benchmark Plus, Bio-Rad, Japan).

### Statistical analysiss

The experiments were carried out in triplicate; data were expressed as mean ± standard error of the mean (SEM). Significant differences in the enzyme assay among each of treated groups at the same time, the cell area of each treated group, CM permeabilization, and MTT assays were analysed by one-way ANOVA followed by Scheffe’s posthoc test. The *p* < 0.01 was considered as the statistically significant difference.

## Results and discussion

### Isolation, purification and identification of α-mangostin

**Fig. 1:**
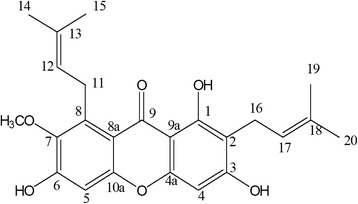
Structure of α-mangostin

**Fig. 2:**
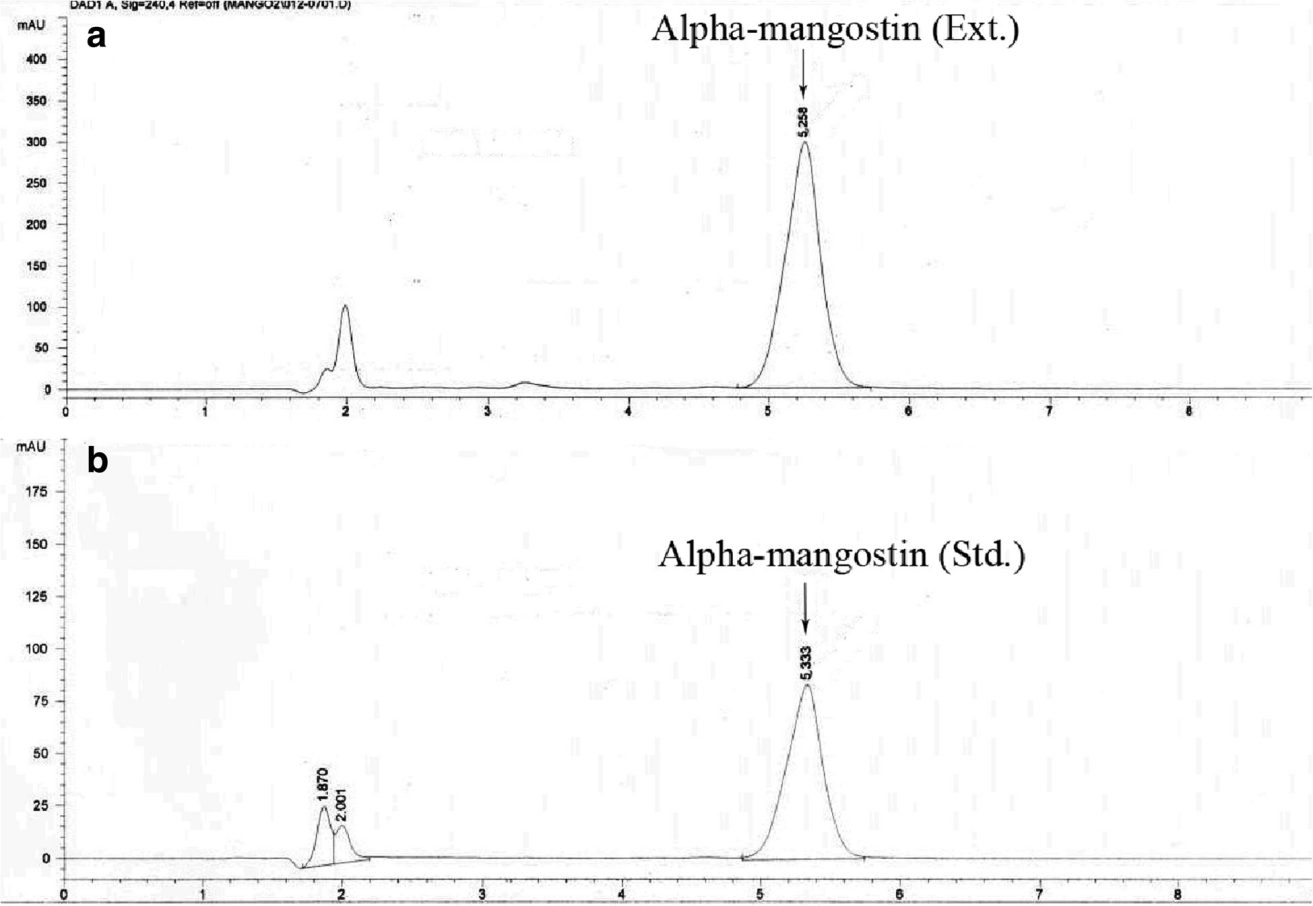
HPLC chromatograms of α-mangostin: **a**, Alpha-mangostin (Ext.) = α-mangostin extract from *G. mangostana*; **b**, Alpha-mangostin (Std.) = α-mangostin standard

The isolated α-mangostin, the percent yield of α-mangostin at 0.016 % (w/w) of dried powder, from the pericarp of GML, was obtained and the chemical structure of α-mangostin from ^1^H NMR and ^13^C NMR (Tables 1 and 2) compared to the reference as illustrated in Fig. 1. Also, the results from HPLC chromatograms of this isolated α-mangostin exhibited a major peak of isolated α-mangostin from the pericarp of GML (Fig. 2a) which is practically the same as a major peak of α-mangostin standard (Fig. 2b). The purity (HPLC) of isolated α-mangostin was 98.0 %.

**Table 1:** The 300 MHz ^1^H NMR (acetone-d6) spectral data of α-mangostin

Chemical Shift (δ, ppm)	Assignment	Chemical Shift (δ, ppm) from reference [15]
13.78	singlet, OH-1	13.72
6.82	singlet, H-5	6.72
6.40	singlet, H-4	6.25
5.27	triplet, H-12, H-17	5.26
4.13	doublet, H-11	4.10
3.80	singlet, 7-OMe	3.78
3.35	doublet, H-16	3.37
2.07	singlet, H-20	1.83
2.05	singlet, H-15	1.82
1.81	singlet, H-14	1.71
1.65	singlet, H-19	1.68

**Table 2:** The 300 MHz ^13^C NMR (acetone-d6), spectral data of α-mangostin

Chemical Shift (δ, ppm)	Assignment	Chemical Shift (δ, ppm) from reference [15]
182.81	C-9	181.8
162.92	C-3	161.6
161.40	C-1	160.2
157.39	C-6	155.4
156.23	C-10a	155.2
155.65	C-4a	154.8
144.51	C-7	142.7
138.14	C-8	137.2
131.39	C-13	131.7
124.82	C-17	123.4
123.50	C-12	122.1
112.06	C-8a	111.7
111.00	C-2	109.7
103.63	C-9a	103.1
102.67	C-5	101.6
93.15	C-4	92.4
61.31	7-OCH_3_	61.2
26.89	C-11	26.3
25.92	C-14	25.7
25.88	C-19	20.7
22.00	C-16	21.3
18.29	C-20	18.1
17.90	C-15	17.7

### MIC and checkerboard determinations

The MIC results for isolated α-mangostin, dissolved in 1 % DMSO, from the pericarp of GML, oxacillin, and nisin against all tested *S. saprophyticus* strains are presented in Table 3. The results indicated that the MICs for isolated α-mangostin, oxacillin, and nisin against all these strains were 8, 128, and 256 μg/ml, respectively. These results indicated that these strains were resistant to oxacillin. Although, the positive control, *Staphylococcus aureus* ATCC 29213, was susceptible to oxacillin at MIC 0.5 μg/ml [32]. The isolated α-mangostin exhibited some inhibitory effect against these strains. These results are in agreement with the studies of Chomnawang et al. and Iinuma et al. who reported antibacterial activity of bioactive compounds from the pericarp of GML extracts against MRSA, *Staphylococcus epidermidis* and *Propionibacterium* strains [13, 33]. Furthermore, previous findings found that the *Garcinia mangostana* extract exhibited MIC values of 0.039 mg/ml against both *Propionibacterium acnes* and *Staphylococcus epidermidis* [34]. The FIC indices for isolated α-mangostin plus oxacillin against all tested *S. saprophyticus* strains were 0.37. These results indicated that these combinations demonstrated synergistic activity against these strains [7, 18]. These findings suggest that isolated α-mangostin from the pericarp of GML extract not only has some antibacterial activity of their own against these strains but also have the ability to reverse the resistance of such bacterial strains by synergy with oxacillin.

**Table 3:** Minimum inhibitory concentration (MIC) of oxacillin, α-mangostin, and nisin alone and in combination

Strains	MIC (μg/ml)			FIC (μg/ml)	FIC index
	OXA	AMT	NIS	OXA + AMT	OXA + AMT
*S. saprophyticus* DMST 27055	128 ^*R*^	8	256	16 + 2	0.37 ^*SI*^
*S. saprophyticus* DMST 27058	128 ^*R*^	8	256	16 + 2	0.37 ^*SI*^
*S. saprophyticus* DMST 4236	128 ^*R*^	8	256	16 + 2	0.37 ^*SI*^
*S. saprophyticus* DMST 4672	128 ^*R*^	8	256	16 + 2	0.37 ^*SI*^
*S. saprophyticus* DMST 5057	128 ^*R*^	8	256	16 + 2	0.37 ^*SI*^
*S. saprophyticus* DMST 8034	128 ^*R*^	8	256	16 + 2	0.37 ^*SI*^
*S. aureus* ATCC 29213^*P*^	0.5 ^*S*^	4	1	N/D	N/D

*S. aureus* ATCC 29213 ^*P*^, was used as a positive control

^*S*^ Susceptible, ^*R*^ Resistant, ^*SI*^ Synergistic interaction, *N/D* Not determine

*OXA* Oxacillin, *AMT* α-mangostin, *NIS* Nisin

The MICs are presented as the median values measured from three independent experiments; each experiment was performed in triplicate

### Kill curve assays

**Fig. 3:**
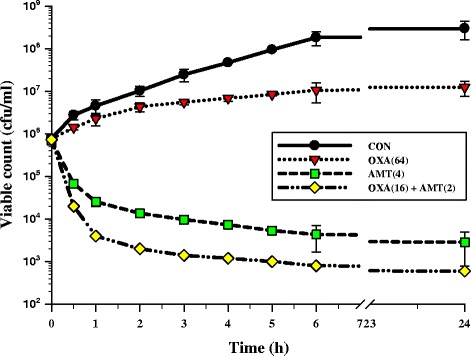
The effect of oxacillin, α-mangostin alone or in combination on the viable counts of ORSS-27055. CON = control (drugs free); OXA(64) = 64 μg/ml oxacillin; AMT(4) = 4 μg/ml α-mangostin; OXA(16) + AMT(2) = 16 μg/ml oxacillin plus 2 μg/ml α-mangostin. The values plotted are the means of 4 observations, and the vertical bars indicate the standard errors of the means

The results for the separate and combined effects of isolated α-mangostin from the pericarp of GML and oxacillin on viable counts of ORSS are presented in Fig. 3. The control showed no reduction in the counts of cfu from control inoculum. The viable counts for the cells treated with isolated α-mangostin at 4 μg/ml were rather lower than that of oxacillin at 64 μg/ml (between 1 and 24 h). Clearly, the combination of 2 μg/ml isolated α-mangostin and 16 μg/ml oxacillin greatly decreased the cell count to 1 × 10^3^ cfu/ml after 4 h to 24 h. These results confirmed the checkerboard assay results, which indicated synergistic activity that the combination produced a decrease of ≥ 2 log10 cfu/ml, compared with oxacillin treatment alone [35]. These results are consistent with those of Eumkeb et al. that galangin plus amoxicillin exhibited synergistic activity against penicillin-resistant *S. aureus* strains at an FIC index of 0.05 [9]. Apart from this, previous findings reported that a synergistic effect using flavonoids and oxacillin against vancomycin-intermediate *S. aureus* showed 87.5 % synergism with FIC indices between 0.0417 - 0.1333 [36].

### Enzyme assays

**Fig. 4:**
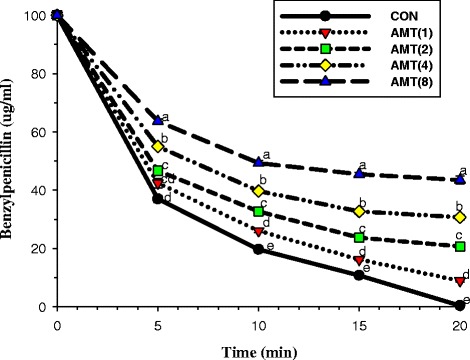
The inhibitory activity of α-mangostin against β-lactamase in hydrolysing benzylpenicillin. β-lactamase used from *E. cloacae*; CON = control (no testing agent); AMT(1) = 1 μg/ml α-mangostin. The graph shows the remaining benzylpenicillin at the same time. Means sharing the same superscript are not significantly different from each other (Scheffe’s test, *p* < 0.01)

The enzyme assay results for the isolated α-mangostin treatment revealed that the levels of benzylpenicillin were significantly higher compared with the controls (*p* < 0.01). The levels of benzylpenicillin depended on the isolated α-mangostin concentration in a dose-dependent manner (*p* < 0.01) (Fig. 4). Previous findings found that 82 % of *Staphylococcus saprophyticus* strains produced β-lactamase [37]. Furthermore, Hirano and Bayer demonstrated that in vivo efficacy of ampicillin plus sulbactam could inhibit oxacillin-resistant *Staphylococcus aureus,* which commonly produce β-lactamase [38]. In addition, previous findings reported that α-mangostin was found to be active against membrane enzymes of *S. mutans* UA159 [39]. Furthermore, These results are in agreement with those of Denny et al. and Zhao et al. that galangin and epigallocatechin gallate, both sharing benzene fused rings condensed with pyran rings (Chromone or 1,4-benzopyrone) similar to α-mangostin, inhibited metallo-β-lactamase and penicillinase, respectively [40, 41]. These findings suggest that the isolated α-mangostin from the pericarp of GML extract could be able to inhibit β-lactamase activity. These inhibitory results may be as a consequence of the isolated α-mangostin form the complex with β-lactamase type IV resulting in deactivation of the β-lactamase activity [9]. Also, the increased of benzylpenicillin was observed in a very short time interval (in minutes; Fig. 9). This result correlates well with the rapid killing of a membrane-targeting antimicrobial. Thus, reduction of the β-lactamase activity could be a secondary effect after the membrane is disrupted [42].

**Fig. 5:**
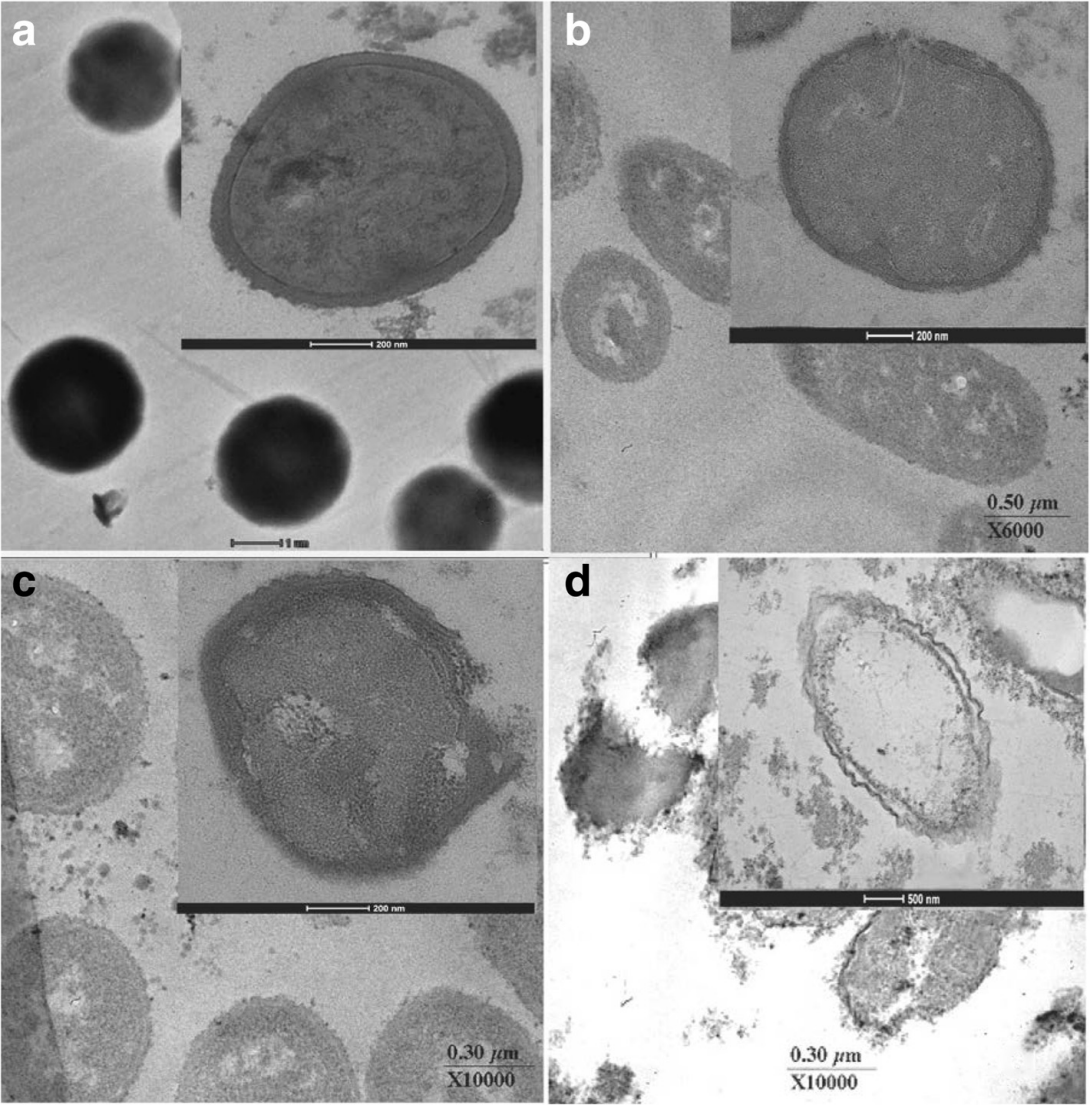
Ultrathin sections of log phase oxacillin-resistant *S. saprophyticus* DMST 27055 grown in CAMHB containing: **a**, control (drug-free); **b**, oxacillin at 64 μg/ml; **c**, α-mangostin at 4 μg/ml; **d**, oxacillin at 12 μg/ml plus α-mangostin at 1.5 μg/ml; (*Magnification*; **a**, 4000×, bar = 1 μm; **b**, 6000×, bar = 0.5 μm; **c,** 10,000×, bar = 0.5 μm; **d**, 10,000×, bar = 0.3 μm; *Inset magnification*; **a**, **c**, 38,000×; **b**, 29,000×; **d**, 10,000×; bar; **a**, **b**, **c**, 200 nm; **d**, 500 nm)

**Fig. 6:**
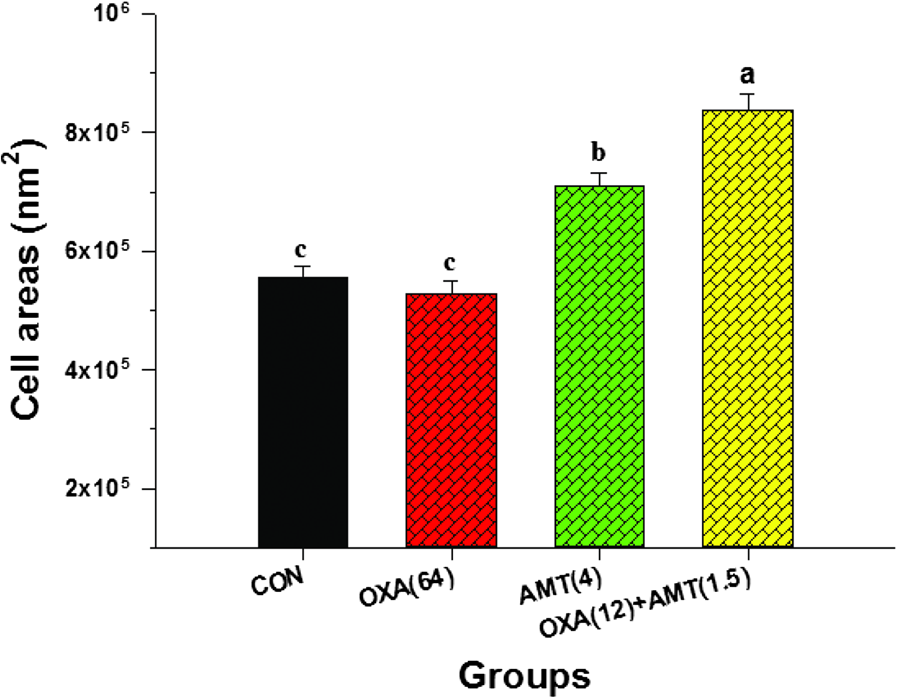
The effect of either oxacillin or α-mangostin on average cross-section of ORSS-27055 cell areas. CON = control (drugs free); OXA(64) = 64 μg/ml oxacillin; AMT(4) = 4 μg/ml α-mangostin; OXA(12) + AMT(1.5) = 12 μg/ml oxacillin plus 1.5 μg/ml α-mangostin. The mean ± SEM for three replicates are illustrated. Means sharing the same superscript are not significantly different from each other (Scheffe’s test, *p* < 0.01)

**Fig. 7:**
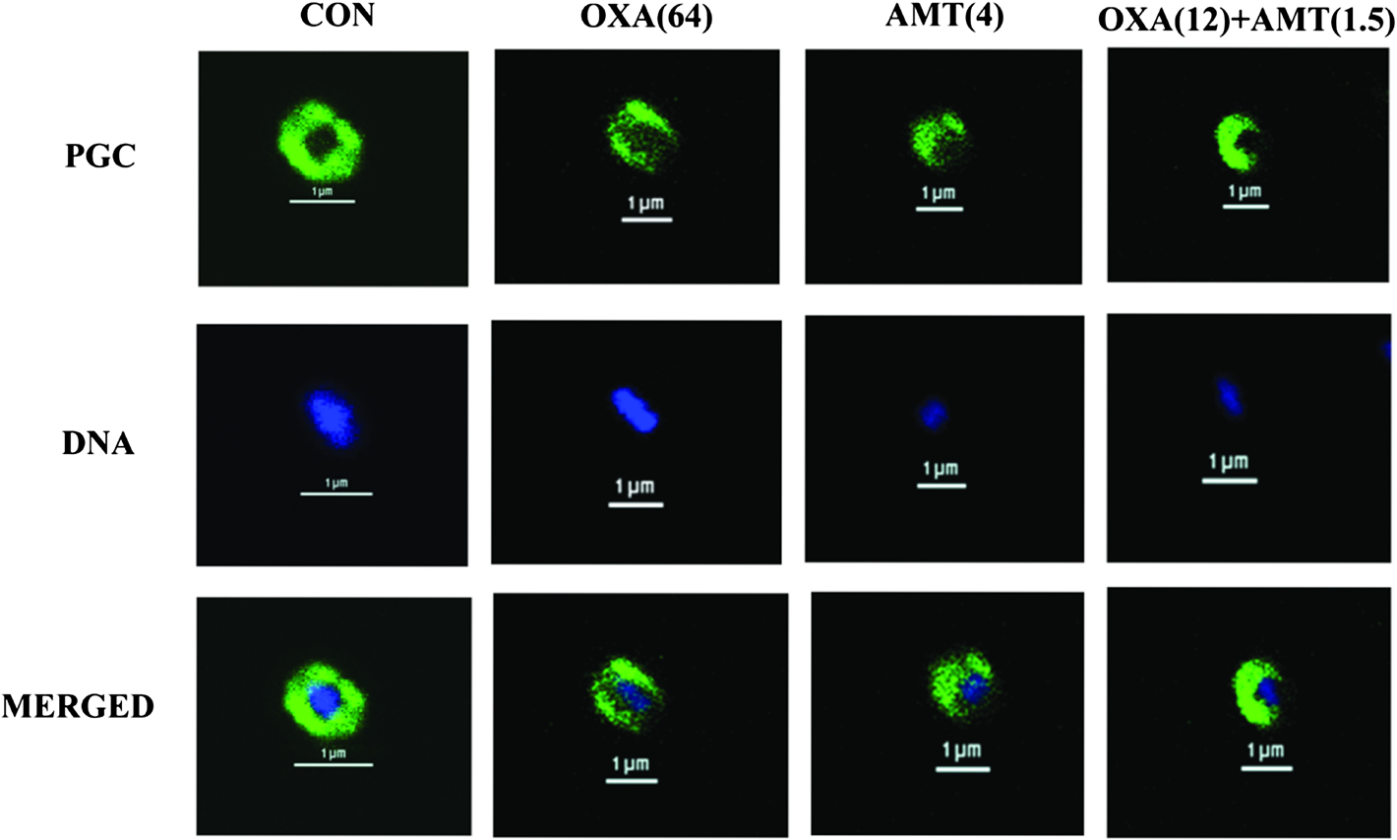
Schematic representation of the results of immunofluorescence and a confocal laser scanning microscope; Samples of oxacillin-resistant *S. saprophyticus* DMST 27055 after treatment for 4 h with oxacillin, α-mangostin either alone or in combination. CON = control (drugs free); OXA(64) = 64 μg/ml oxacillin; AMT(4) = 4 μg/ml α-mangostin; OXA(12) + AMT(1.5) = 12 μg/ml oxacillin plus 1.5 μg/ml α-mangostin. The cells were stained for DNA with DAPI (blue, DNA) and labelled for peptidoglycan (green, PGC) using respective antibodies. DNA in all groups was localized in the central of the cell and surrounded by a peptidoglycan layer (MERGED). Scale bar = 1 μm

**Fig. 8:**
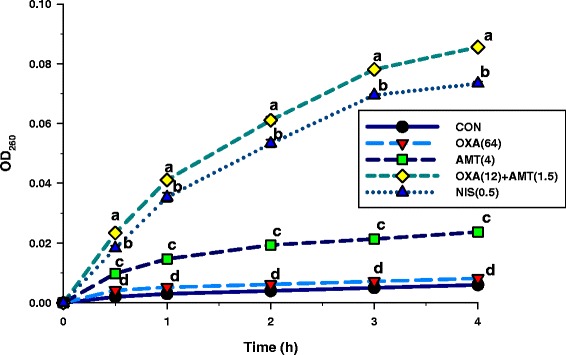
Effects of 260 nm absorbing material (DNA, RNA, and metabolites) in the ORSS-27055 supernatants. These cells were treated with oxacillin, α-mangostin either alone or in combination. CON = control (drugs free); OXA(64) = 64 μg/ml oxacillin; AMT(4) = 4 μg/ml α-mangostin; OXA(12) + AMT(1.5) = 12 μg/ml oxacillin plus 1.5 μg/ml α-mangostin; NIS(0.5) = 0.50 μg/ml nisin. Nisin at 0.50 μg/ml was used as a positive control, and untreated cells were used as a negative control. The mean ± SEM for three replicates are illustrated. Means sharing the same superscript at the same time are not significantly different (Scheffe’s test, *p* < 0.01)

**Fig. 9:**
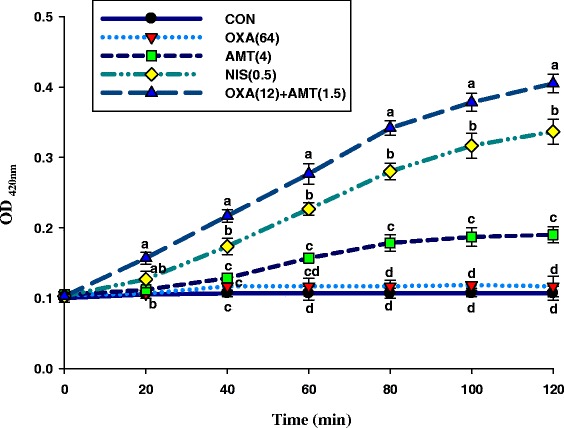
Time-dependency of oxacillin plus α-mangostin-induced permeabilisation of the cytoplasmic membrane of ORSS-27055. The kinetics of α-mangostin-mediated ONPG passage across the cytoplasmic membrane are monitored as a change in the optical density (OD). The concentration of substrates was 100 μg/ml ONPG. CON = control (drugs free); OXA(64) = 64 μg/ml oxacillin; AMT(4) = 4 μg/ml α-mangostin; OXA(12) + AMT(1.5) = 12 μg/ml oxacillin plus 1.5 μg/ml α-mangostin; NIS(0.5) = 0.50 μg/ml nisin. Nisin at 0.50 μg/ml was used as a positive control, and untreated cells were used as a negative control. Each treatment performed two times in triplicate. The graph shows OD_420nm_ of each treatment at the same time. Means sharing the same superscript at the same time are not significantly different from each other (Scheffe’s test, *p* < 0.01)

### TEM

The transmission electron micrographs of cells from the log phase of growth of ORSS in the presence of isolated α-mangostin, oxacillin either alone and in combination are presented in Fig. 5. The untreated cells appeared normal, peptidoglycan, and cytoplasmic membranes were clearly seen intact. And the morphology of the cells looked normal (Fig. 5a). The ORSS cells treated with oxacillin are displayed in Fig. 5b. This result showed some disruption to both peptidoglycan and cytoplasmic membrane. The average cross-sectional cell areas of these cells were slightly smaller than the controls, but there was no significant difference (*p* > 0.01) (Fig. 6). Although, the micrograph of these cells after exposure to isolated α-mangostin alone is shown in Fig. 5c. Clear damage to peptidoglycan and the cytoplasmic membrane was evident. The average cell areas of these cells were significantly bigger than those of controls (*p* < 0.01) (Fig. 6). Fig. 5d reveals the isolated α-mangostin plus oxacillin-treated cells. These cells displayed the greatest damage to the peptidoglycan and cytoplasmic membrane resulting in leakage of intracellular materials and overall morphological changes. Clearly, these average cell areas were significantly bigger than the control (*p* < 0.01) (Fig. 6). These results provide evidence that isolated α-mangostin shows stronger activity than oxacillin against this strain at these concentrations. These findings agree with previous findings where the combination of ceftazidime plus galangin led to damage of the cell ultrastructures, the integrity of cell walls and increase in cell size of ceftazidime-resistant *S. aureus* [9]. Furthermore, this current study shows similarity to that of the work of Koh et al. who reported that α-mangostin caused significant morphological effects to *S. aureus* (MRSA) including wall damage and cell lysis [42].

### Immunofluorescence staining and confocal microscopy

The peptidoglycan and DNA-labelled ORSS clearly showed intact coccus-shape and no damage was observed in untreated control cells by confocal laser scanning images (Fig. 7). The cells treated with isolated α-mangostin or oxacillin alone displayed minor peptidoglycan damage and DNA leakage. The combination of these agents caused considerable peptidoglycan damage and DNA leakage compared to controls. The merger of peptidoglycan and DNA images are also shown. These results are in substantial agreement with the TEM study and support a preliminary mechanism of action of this combination being targeted at the peptidoglycan structure.

### CM permeabilization

The CM permeability was measured by examining the release of UV-absorbing materials at 260 nm (Fig. 8). After treatment, ORSS cells with isolated α-mangostin, nisin, and the isolated α-mangostin plus oxacillin combination could induce the release of 260 nm absorbing materials at significantly higher levels compared with the control or oxacillin alone (*p* < 0.01). The CM permeabilising ability was ranked as follows isolated α-mangostin plus oxacillin > nisin > isolated α-mangostin > oxacillin > control (*p* < 0.01). These results suggested that the synergistic activity of isolated α-mangostin plus oxacillin resulted in increased cytoplasmic membrane permeability of DNA, RNA, and cellular metabolites [26, 27].

The α-mangostin-induced CM permeabilisation of the ORSS by ONPG uptake results are shown in Fig. 9. The ORSS cytoplasmic membrane was permeabilised much more rapidly by the isolated α-mangostin plus oxacillin compared to other groups. Nisin, which is highly active against the outer membrane, showed CM permeability significantly lower than isolated α-mangostin and oxacillin combination (*p* < 0.01). These results are consistent with the results of CM permeabilisation with the UV-absorbing material at 260 nm (Fig. 8). In the same way, previous studies found that galangin, which shares the chromone (or 1,4-benzopyrone) structure with α-mangostin, caused CM permeabilisation of *S. aureus* resulting in potassium loss [9, 43].

Our findings lend support to previous findings that α‐mangostin rapidly disrupted the integrity of the cytoplasmic membrane of MRSA cells, leading to losing of intracellular components in a concentration-dependent manner [42]. The CM permeability results provide evidence that one of the important mechanism of action of isolated α-mangostin is disruption of the cytoplasmic membrane. This disruption in turn leads to deactivation of the β-lactamase activity.

Plant-derived antibacterial compounds have weaker antibacterial activity compared to that of synthetic antibiotics. Therefore, synergistic paradigms by combining the conventional antibiotic with phytochemical compounds is proven in several studies to be an effective avenue to treat infectious diseases caused by drug-resistant bacteria [7–9]. Synergistic interaction combats drug-resistant bacteria by achieving multiple synergistic drug targets, interacting with drug-resistant mechanisms of bacteria, and neutralising and eliminating adverse effects [7].

Previous studies found that ceftazidime had synergistic activity with baicalein, luteolin or quercetin, which shared Chromone structure, against *Streptococcus pyogenase* [23, 44]. In the same way, Eumkeb and co-workers reported that certain β-lactam drugs plus galangin, quercetin or baicalein, which also shared chromone structure, showed synergistic activity against penicillin-resistant *S. aureus* [9]. Besides, Rukayadi et al. found that Panduratin A, which possesses a benzene ring and an isoprenyl group, displayed an MIC of 1 μg/ml for staphylococcal clinical isolates and generally was more potent than commonly used antimicrobials [45]. Also, molecular dynamic simulations revealed that isoprenyl groups of α-mangostin, which occupy chromone structure and isoprenyl groups, played an important role in penetrating the lipid bilayer of the MRSA membrane resulting in membrane breakdown and increased permeability [42]. These findings provide evidence that the benzene ring and the isoprenyl group of both panduratin A and α-mangostin play a significant role in inhibiting the growth of MRSA strains by s direct interactions with the bacterial membrane [42, 45].

### In vitro cytotoxic test (MTT assays)

**Fig. 10:**
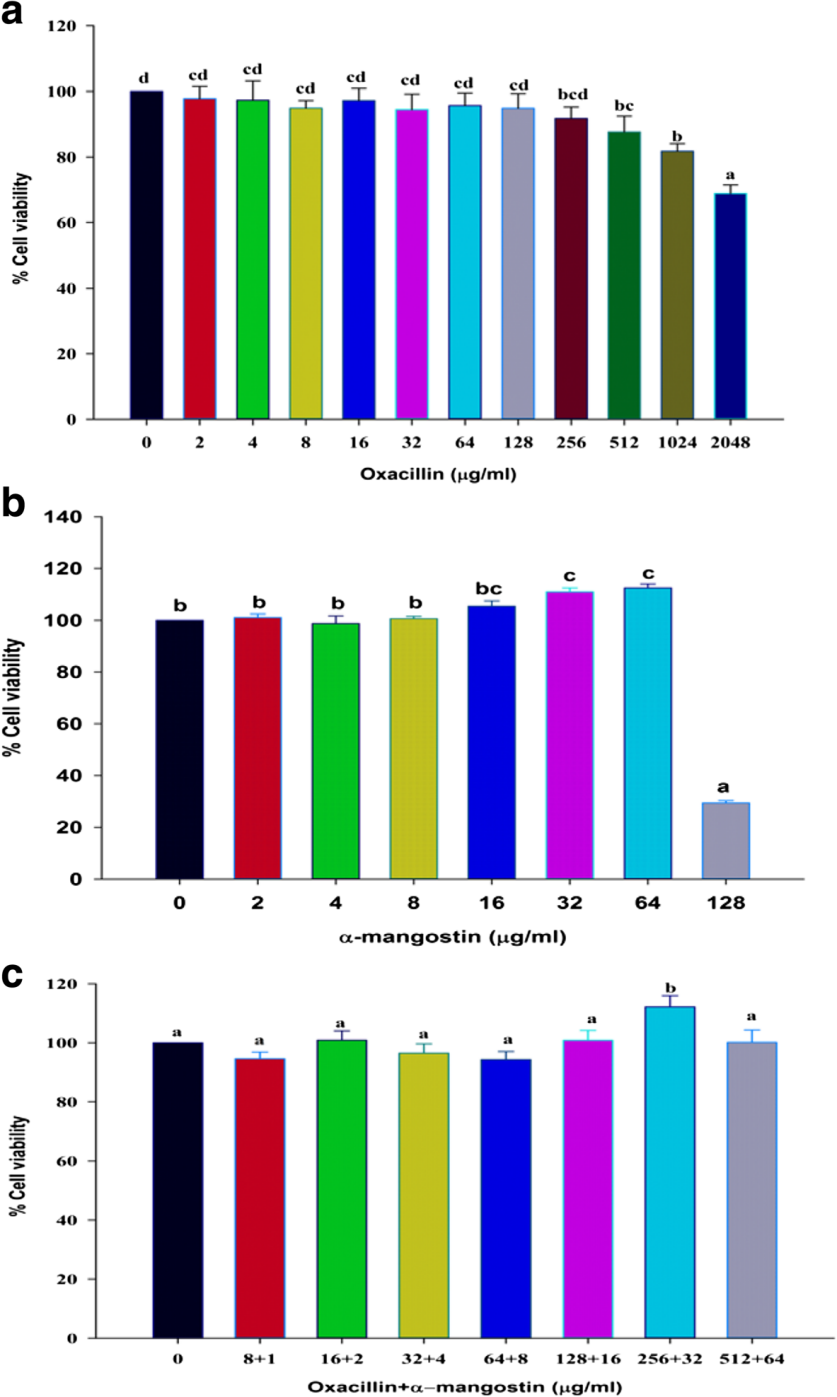
The effect of oxacillin, α-mangostin either alone or in combination on 3T3-L1 preadipocytes. 3T3-L1 preadipocytes were treated with: **a**. oxacillin (0-2048 μg/ml); **b**. α-mangostin (0-128 μg/ml); and **c**. oxacillin plus α-mangostin (8 + 1 to 512 + 64 μg/ml), for 24 h. Results are expressed as percentages of cell viability as compared with untreated controls (*n* = 8). The mean ± SEM for three replicates are illustrated. Means sharing the same superscript are not significantly different from each other (Scheffe’s test, *p* < 0.01)

The results of the MTT assays are shown in Fig. 10. The α-mangostin or oxacillin alone initially exhibited cytotoxicity against 3T3-L1 preadipocytes at concentrations 128 and 1024 μg/ml, respectively, which is 16 and 8 times higher than the MICs of α-mangostin and oxacillin against *S. saprophyticus* respectively (Fig. 10a and b). Moreover, Fig. 10c revealed the combination of α-mangostin and oxacillin at concentrations of 2 and 16 μg/ml, which showed the synergistic effect of antibacterial activity against this strain, had no cytotoxic effect towards 3T3-L1 preadipocytes after 24 h of exposure. Moreover, the four times higher than FICI value, 64 μg/ml oxacillin plus 8 μg/ml α-mangostin, still did not show the cytotoxic effect on 3T3-L1 preadipocytes. Our findings provide evidence that the MICs and higher dosages of agents have not shown a cytotoxic effect on this cell line. Importantly, a greatly desired property of antibacterial compounds is the selective inhibition against bacterial with less cytotoxic effect to normal cells for avoiding side effects to healthy tissues [46]. The previous studies reported that the IC_50_ of α-mangostin on the MRC-5 cell line is at the concentration of 7.5 μM [47] and 50 μM on 3T3-L1 preadipocyte cell line [48]. These findings imply that α-mangostin used alone or in combination may be useful in developing a novel adjunct phytopharmaceutical to oxacillin for the treatment of ORSS.

## Conclusions

In conclusion, our findings provide evidence that isolated α-mangostin from the pericarp of GML alone has not only some activity against ORSS but also possesses the synergistic activity with oxacillin against this strain. The chromone (or 1,4-benzopyrone) structure and isoprenyl groups of α-mangostin could play an important role in inhibiting this strain. This synergistic activity of isolated α-mangostin plus oxacillin may involve three modes of action of this xanthone. Firstly, potential effects of cytoplasmic membrane disruption and increases permeability. Secondly, inhibition of β-lactamase activity. Finally, peptidoglycan damage. Our findings provide evidence that isolated α-mangostin from the pericarp of GML has a sufficient margin of safety for therapeutic use. Isolated α-mangostin provides potential to develop a useful of novel adjunct phytopharmaceutical to oxacillin for the treatment of ORSS. Future studies should address toxicity tests in animals and humans.

## Abbreviations

CAMHB: Cation-adjusted Meuller-Hinton broth
CM: Cytoplasmic membrane
FIC: Fraction inhibitory concentration
FICI: Fraction inhibitory concentration index
GML: *Garcinia mangostana* L.
MHA: Meuller-Hinton agar
MHB: Meuller-Hinton broth
MIC: Minimum inhibitory concentration
ONPG: O-nitrophenol-β-D-galactoside
ORSS: Oxacillin-resistant *Staphylococcus saprophyticus*
ORSS-20755: Oxacillin-resistant *Staphylococcus saprophyticus* DMST 20755
TEM: Transmission electron microscopy.
